# COPD immunopathology

**DOI:** 10.1007/s00281-016-0561-5

**Published:** 2016-05-13

**Authors:** Gaetano Caramori, Paolo Casolari, Adam Barczyk, Andrew L. Durham, Antonino Di Stefano, Ian Adcock

**Affiliations:** 1 Centro Interdipartimentale per lo Studio delle Malattie Infiammatorie delle Vie Aeree e Patologie Fumo-correlate (CEMICEF; formerly named Centro di Ricerca su Asma e BPCO), Sezione di Medicina Interna e Cardiorespiratoria, Università di Ferrara, Via Savonarola 9, 44121 Ferrara, Italy; 2 Katedra i Klinika Pneumonologii, Slaski Uniwersytet Medyczny w Katowicach, Katowice, Poland; 3 Airways Disease Section, National Heart and Lung Institute, Imperial College London, London, UK; 4 Divisione di Pneumologia e Laboratorio di Citoimmunopatologia dell’Apparato Cardio Respiratorio, Salvatore Maugeri Foundation, IRCCS, Veruno, NO Italy

## Abstract

The immunopathology of chronic obstructive pulmonary disease (COPD) is based on the innate and adaptive inflammatory immune responses to the chronic inhalation of cigarette smoking. In the last quarter of the century, the analysis of specimens obtained from the lower airways of COPD patients compared with those from a control group of age-matched smokers with normal lung function has provided novel insights on the potential pathogenetic role of the different cells of the innate and acquired immune responses and their pro/anti-inflammatory mediators and intracellular signalling pathways, contributing to a better knowledge of the immunopathology of COPD both during its stable phase and during its exacerbations. This also has provided a scientific rationale for new drugs discovery and targeting to the lower airways. This review summarises and discusses the immunopathology of COPD patients, of different severity, compared with control smokers with normal lung function.

## Introduction

**Fig. 1:**
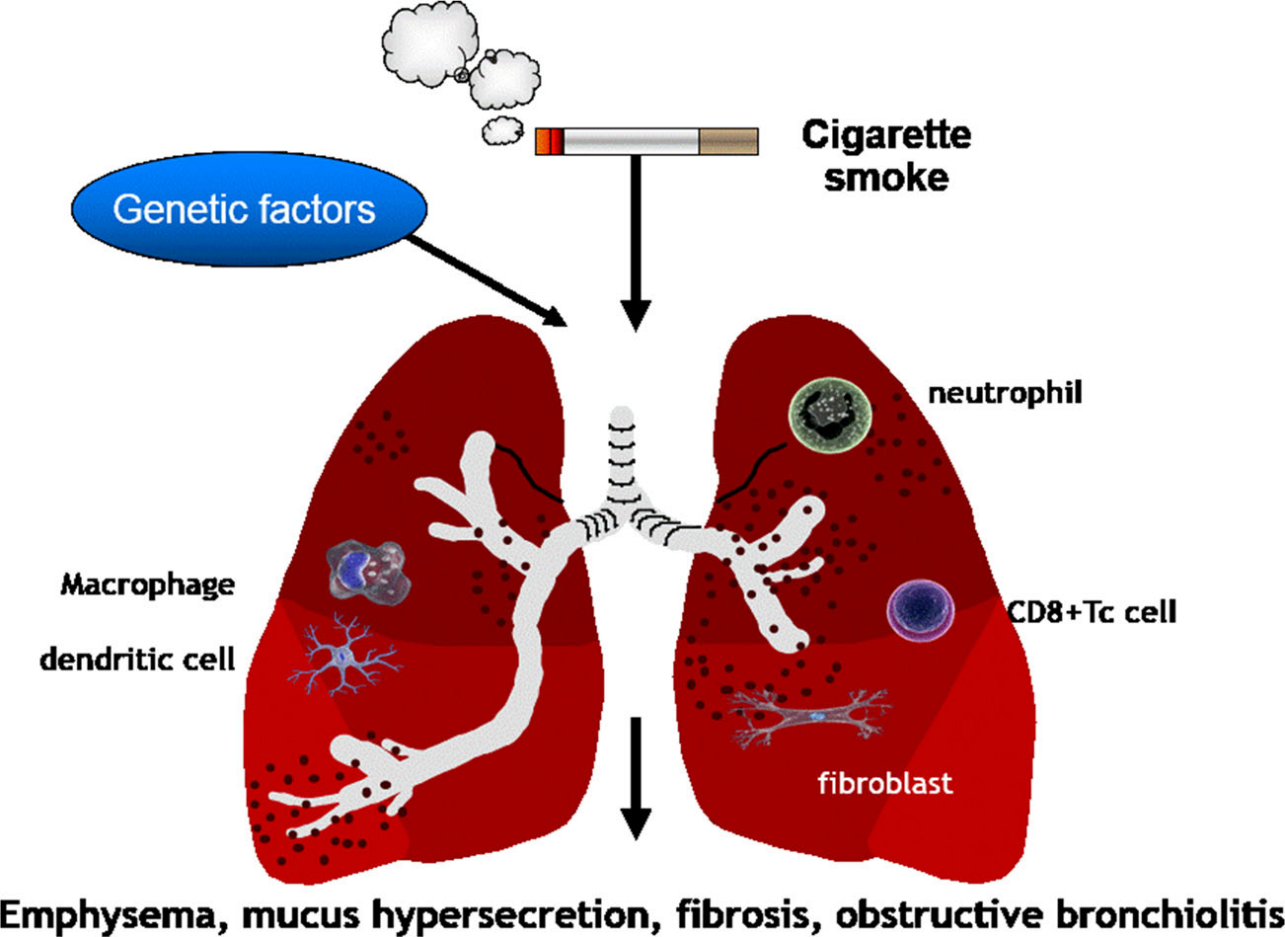
In the Western world, COPD is mainly related to cigarette smoking. The cigarette smoke activates macrophages, dendritic cells and airway epithelial cells in response to toxic particles in the smoke. Once activated, these cells release mediators that recruit and activate CD8+ T-lymphocytes (CD8+ Tc cells) and neutrophils. The inflammatory process also mediates small airway fibrosis. The activation of these and other cell types and the activation of inflammatory and remodelling processes lead to small airway fibrosis, obstructive bronchiolitis, pulmonary emphysema and mucus hypersecretion

Chronic obstructive pulmonary disease (COPD) is the 3rd leading cause of morbidity and mortality worldwide [1]. It is defined as “a common preventable and treatable disease, characterised by persistent airflow limitation that is usually progressive and associated with an enhanced chronic inflammatory response in the airways and the lung to noxious particles or gases. Exacerbations and comorbidities contribute to the overall severity in individual patients” [1]. The etiology of COPD is due to complex interactions between environmental factors (particularly cigarette smoking) and genetic factors. Long-term cigarette smoking is currently the cause of more than 90 % of COPD in Westernised countries (Fig. 1) whereas other factors, such as burning biomass fuels for cooking and heating, may be important causes of COPD in developing countries [1, 2]. Only ∼25 % of chronic smokers develop symptomatic COPD by the age of 80 years, suggesting a genetic component, but the influence of single gene polymorphisms is weak [3] and the only clearly established, albeit rare, genetic risk factor for COPD is α_1_-antitrypsin deficiency (α_1_-AT) [4].

There are, so far, very few studies comparing the pathology between cigarette-smoking-associated COPD and other causes of disease [5, 6]; for this reason, our review of the literature will be limited to the immunopathology in cigarette-smoking-associated COPD. The progressive chronic airflow limitation in COPD is due to two major pathological processes: remodelling and narrowing of small airways and destruction of the lung parenchyma with consequent loss of the alveolar attachments of these airways as a result of pulmonary emphysema. This results in diminished lung recoil, higher resistance to flow and closure of small airways at higher lung volumes during expiration, with consequent air trapping in the lung. This leads to the characteristic hyperinflation of the lungs, which gives rise to the sensation of dyspnea and decrease exercise tolerance [7]. Both the small-airway remodelling and narrowing and the pulmonary emphysema are likely to be the results of chronic inflammation in the lung periphery [8].

The major site of increased resistance is localised to the small airways less than 2 mm in internal diameter, which are located from the 4th to the 12th generation of airway branching in the lung] [9–11] and was confirmed using 3-D computed tomography [12]. Around 80 % of the conducting airways beyond this point are nonrespiratory bronchioles, and the remaining 20 % are smaller bronchi identified by the presence of cartilage plaques in their walls [13]. Bronchioles differ from bronchi by having no cartilage and submucosal glands, a relatively greater proportion of smooth muscle and fewer mucus-secreting cells in the epithelial layer. In normal subjects the small airways have a much larger collective cross-sectional area compared with the central airways so that physiologically they contribute only around 20 % of total airflow resistance. This is the reason why more of 80 % of the small airways need to be occluded before there is any demonstrable airflow impairment and why many cigarette smokers develop a progressive small-airway disease long before airflow obstruction is detected [14].

## COPD immunopathology in stable patients

### Inflammatory cells in the wall of lower airways of stable COPD patients

Inflammation is a central feature of stable COPD causing activation and alteration in the structural cells of the airways and lungs (lower airways and lung remodelling) and the activation and/or recruitment of infiltrating inflammatory cells [15–18]. The chronic airflow obstruction in smoking-induced COPD results from a combination of small-airway inflammation and remodelling and loss of lung elasticity due to lung parenchymal destruction. Although pulmonary emphysema usually only appears with increasing disease severity, it can also occur in subjects without airflow obstruction [17, 19–21]. Lesions in the small airways are a major determinant of COPD progression and severity, and there is a strong inverse association between total small airway wall thickness and FEV_1_ [8].

**Fig. 2:**
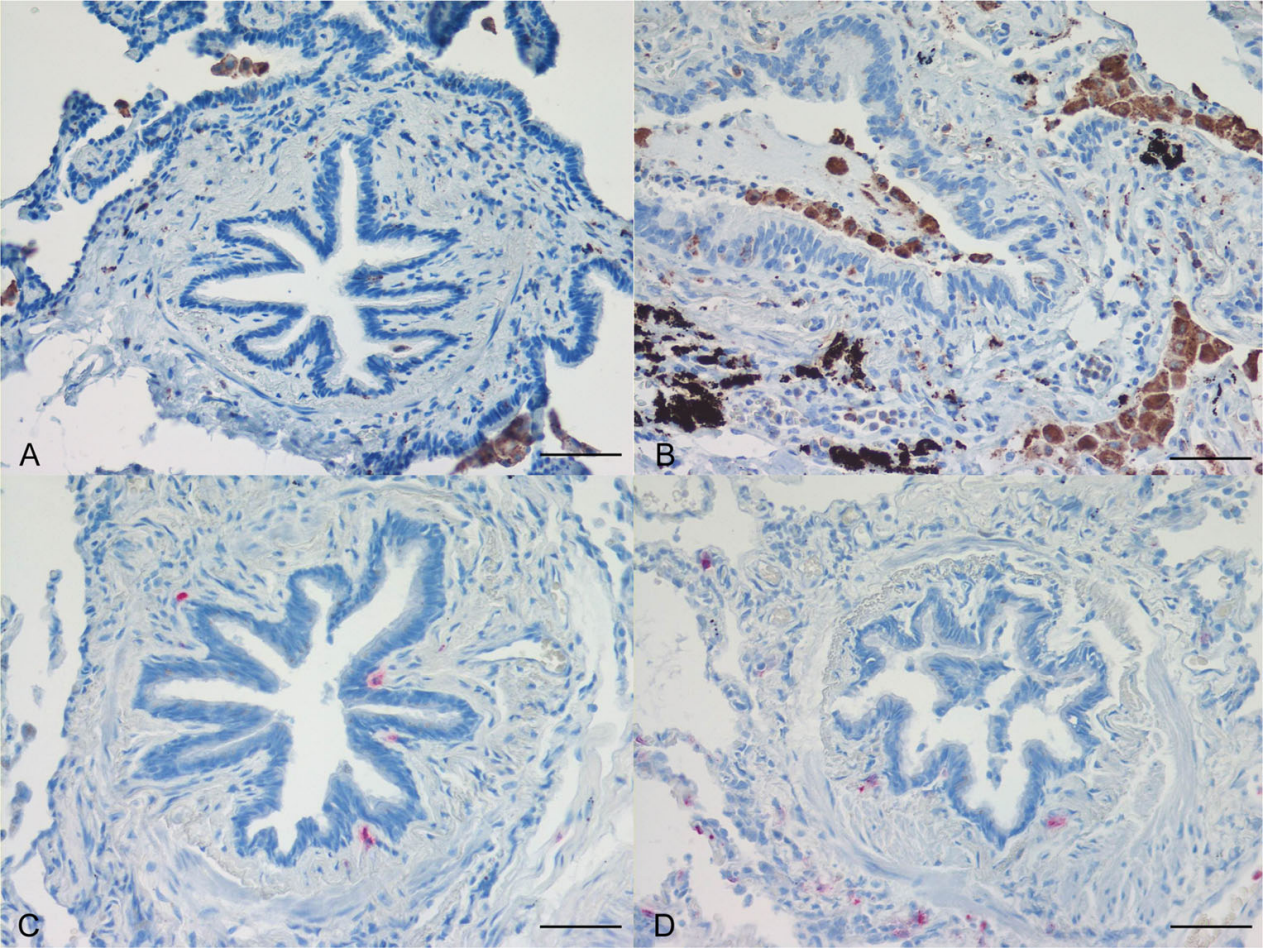
Representative immunohistochemical staining for CD68 (*DAB*; *brown*) of alveolar macrophages (**a**, **b**) and for neutrophil elastase (alkaline phosphatase, *red*) (**c**, **d**) in paraffin sections of the small airways of stable moderate COPD patients (**b**, **d**) and of control smokers with normal lung function (**a**, **c**). Pictures total magnification = ×200; *bar* = 50 μm

Lower airways and lung inflammation in stable COPD patients is characterised by increased numbers of macrophages, neutrophils, T and B lymphocytes and dendritic cells (Fig. 2). However, the predominant inflammatory cell type varies with disease severity; increased numbers of neutrophils and B lymphocytes are present in the most severe (grades III and IV) disease [8, 16–18]. The functional role of these inflammatory cells, including their subsets, is still largely unknown but the interaction between lymphocytes and macrophages may orchestrate the onset, progression and severity of lower airways inflammation and there is mounting evidence that innate immunity, but not inflammasome activation, correlates with the progression of the severity of stable COPD [22].

Analysis of inflammatory cell infiltration in bronchial biopsies of patients with stable mild/moderate COPD shows an increased inflammatory cell infiltration in comparison with control nonsmokers [15, 17, 18]. In smokers, the development of airflow obstruction is associated with more pronounced small-airway inflammation and appearance of increased thickness of their wall due to fibrosis and smooth muscle hypertrophy [15, 17, 18]. There is a regional distribution of the inflammatory process in the small airways of patients with COPD. Smokers with COPD have a greater density of leukocytes (CD45+ cells) in the submucosa (which extends from the distal edge of the basement membrane to the internal edge of the smooth muscle) compared with the adventitia (which extends from the outer edge of the smooth muscle to the alveolar attachments) [23].

Hogg and colleagues assessed the small airways (inner diameter less of 2 mm) in surgically resected lung tissue from 159 patients (39 with stage 0 (smokers with normal lung function and chronic bronchitis), 39 with stage 1 (mild), 22 with stage 2 (moderate), 16 with stage 3 (severe) and 43 with stage 4 (very severe) COPD, according to the 2004 GOLD classification [1, 8, 11]. This study clearly demonstrated that lesions in the small airways are a major determinant of the progression and severity of COPD. In fact, there is a strong inverse association between total wall thickness, measured as the ratio of the volume to the surface area (V/SA), in the small airways and FEV_1_ [8, 11].

Progression of COPD is also associated with the accumulation of inflammatory mucous exudates in the lumen and infiltration of the wall by innate and adaptive inflammatory immune cells that form lymphoid follicles [8, 11]. The percentage of the small airways that contain CD4+ cells, CD8+ cells, B cells, lymphoid aggregates containing follicles, macrophages and neutrophils, also increased as the severity of COPD progressed compared with control smokers with normal lung function [8, 11]. For this reason, the inflammatory response present in the small airways of the patients with stable COPD is considered an amplification of the inflammatory response to irritants that is seen in smokers with normal lung function [10, 24].

### T lymphocytes and COPD immunopathology in stable patients

Most studies have found an increased number of CD8+ T lymphocytes, in the blood and lower airway tissues of patients with mild/moderate stable COPD; these cells are also increased in the sputum and BAL, but in these secretions, the number of lymphocytes is negligible and very difficult to count. Smoking status, smoking history, degree of airflow obstruction and pulmonary emphysema are all related to increased CD8+ cells and/or CD8+/CD4+ ratio [15, 18]. The number of these activated CD4+, CD8+ cells expressing nuclear factor-kappa B (NF-κB), STAT-4, interferon (IFN)-gamma and perforin was also increased [18]. The functional role of CD3+ (and their subsets CD4+ and CD8+) T lymphocytes in the immunopathogenesis of COPD is scarcely known, and it is an area of active research.

The number of sputum CD8+ cells is also increased in stable COPD patients compared with control smokers with normal lung function and nonsmoking subjects [25], and these are highly activated, expressing high levels of perforin [26]. Sputum CD8+-interleukin (IL)-4 cells are reduced both in stable COPD patients and in control smokers with normal lung function compared with control nonsmoking subjects, while CD8+-IFN-gamma cells are significantly reduced only in COPD as compared with controls. A significant relationship between the CD8+-IL-4/CD8+-IFN-gamma ratio and FEV1 (% pred) is found only in COPD patients [25].

T lymphocytes, mainly CD8+ cells, predominate in the bronchial mucosa of stable COPD patients [15, 17, 18]. However, when mild/moderate COPD patients are compared with control smokers with normal lung function, matched for age and smoking habit, there are no differences in the numbers of CD3+ and CD8+ cells in the submucosa [27]. Smokers with normal lung function also showed, though to a lesser extent, increased numbers of CD3+ and CD8+ cells compared with control nonsmokers [27, 28]. These data suggest that the T lymphocyte increases may be an effect of smoking [29].

**Fig. 3:**
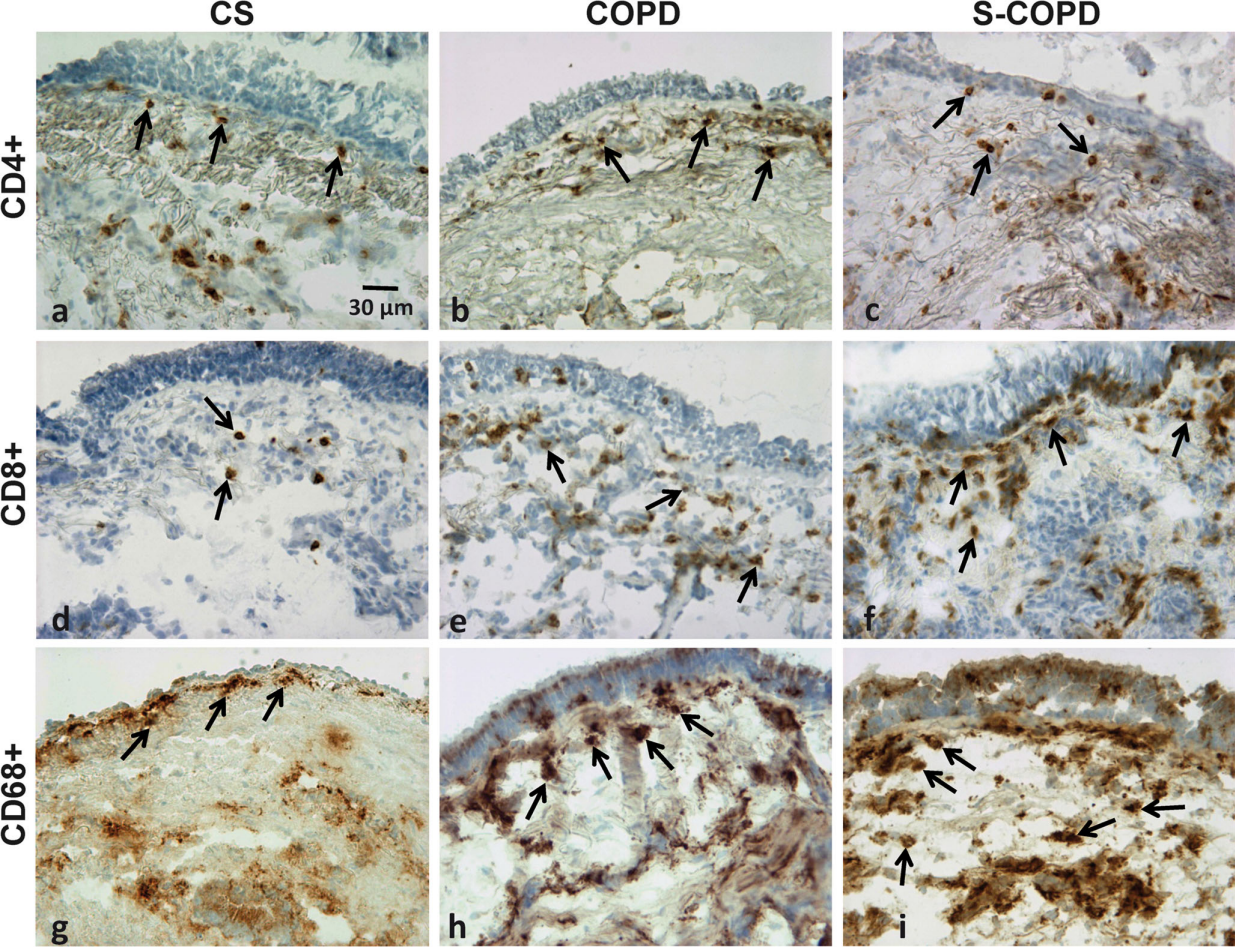
Representative immunohistochemical staining showing the expression and localisation of CD4+ T cells (**a**–**c**), CD8+ T cells (**d**–**f**) and CD68+ macrophages (**g**–**i**) in the airways. The differences in expression between smokers with normal lung function (*CS*; **a**, **d** and **g**), mild/moderate COPD subjects (*COPD*; **b**, **e** and **h**) and in severe COPD patients (*S-COPD*) are also shown. *Arrows* indicate the positively stained cells

Most studies initially reported no significant changes in the number of CD4+ and CD8+ T lymphocytes (or in their ratio) in the small-airway wall of patients with mild/moderate stable COPD compared with control smokers with normal lung function. More recently, however, Hogg and co-workers reported that the number of CD4+ and CD8+ in the small airways increases as the severity of COPD progress compared with control smokers with normal lung function [8] (Fig. 3). The reduced apoptosis of CD8+ T lymphocytes may be an important mechanism that contributes to the accumulation of these cells in the airway submucosa in smokers with mild/moderate COPD [30]. These cells are active and more mature since 30 % of CD4+ and CD8+ T lymphocytes co-expressed the nuclear p65 NF-κB subunit in the bronchial biopsies of COPD patients [27], and in patients with stable COPD, BAL CD8+CD45RA+ and lower CD8+CD45R0+ number are seen than in smokers with normal lung function [31]. Mature T cells have a greater propensity to cause tissue damage [31].

### Th/Tc1, Th2/Tc2, Th17, and γδ T cells lymphocyte subsets and COPD immunopathology in stable patients

**Fig. 4:**
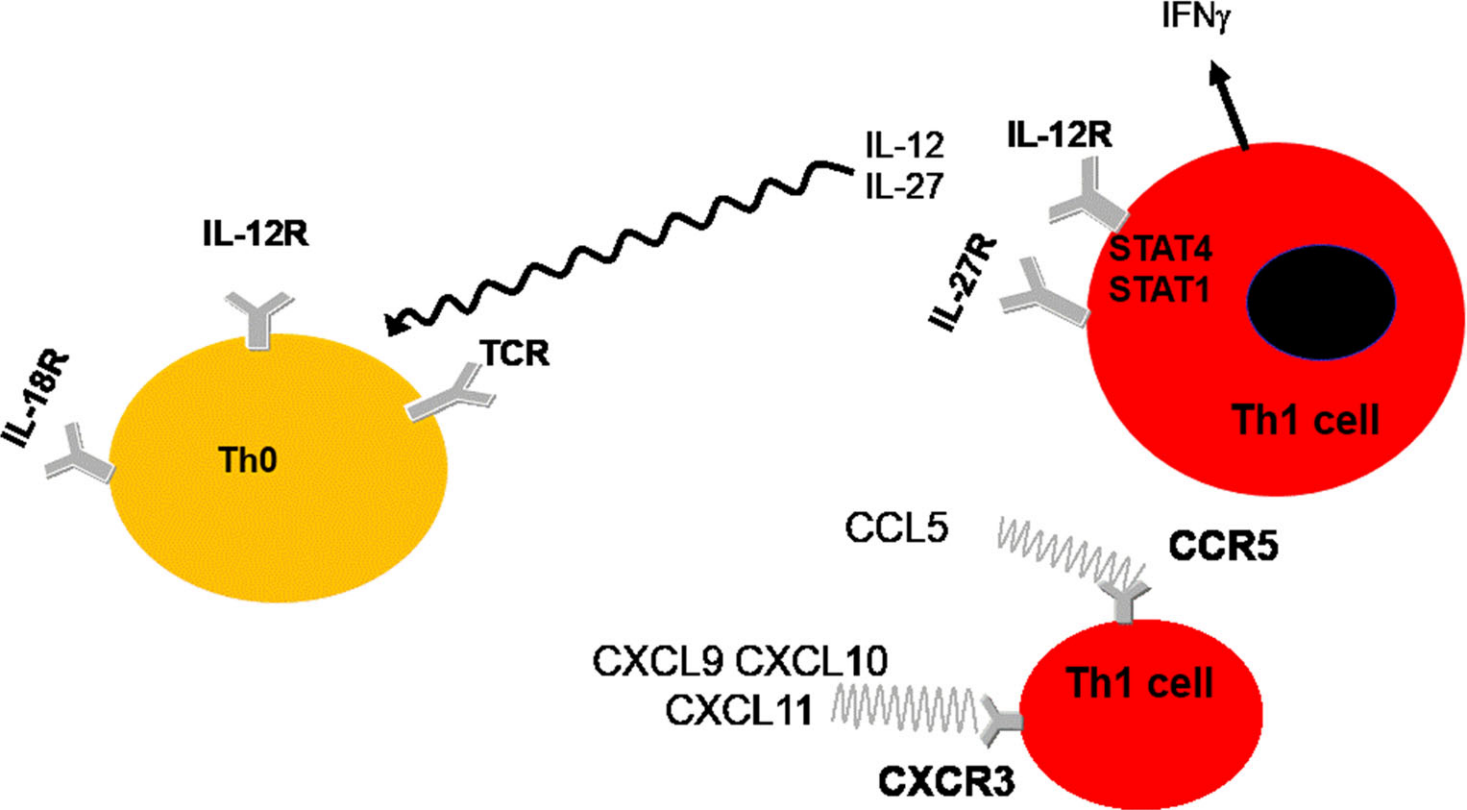
Interleukin 12 (IL-12) acts on IL-12R localised on Th0 to drive Th1 polarisation in conjunction with activation of the IL-18R and the T cell receptor (*TCR*). IL-12 and IL-27 can also act on their receptors on Th1 cells to elicit the expression of interferon (IFN)-γ via STAT activation. The activation status of Th1 cells is also regulated by the CCL5/CCR5 and CXCL9, CXCL10 and CXCL11/CXCR3 axis

The chemokine receptor CCR5, preferentially expressed by Th1/Tc1 cells producing IFN-γ, was reported to be increased in mild/moderate disease in comparison with control smokers [28], suggesting that, at variance with asthma, a prevalent Th1/Tc1 immunosurveillance develops in mild/moderate COPD patients in stable conditions (Fig. 4). This was supported by the enhanced expression of the transcription factor signal transducer and activators of transcription (STAT)4 in the bronchial epithelium and submucosa of mild/moderate COPD patients in comparison with both control smokers and nonsmokers [18]. STAT4 nuclear expression correlated significantly with the number of IFN-γ+, CD3+ and CD4+ cells but not with CD8+ cells in the submucosa of smokers: 50 % of CD4+, one third of CD8+ and one third of CD68+ cells in the bronchial submucosa co-expressed the STAT4 protein nonsmokers [18]. These data reinforce the notion of a prevalent Th1/Tc1 immunological response in mild/moderate disease. At the same time, STAT4 will release the hold on T cell effector function as a result of CTLA‐4 allowing the T cells to become aggressive effectors with the full potential of causing lung injury [32].

The demonstration that the production of IL-4 in the glands of chronic bronchitics is independent of CD8+ lymphocytes [33], again suggests that CD4+ lymphocytes could be mainly involved in the Th2 cytokine response associated with hypersecretion by mucous glands in smokers. These data highlight the importance of activation status over cell presence.

The T lymphocyte subset Th17, producing IL-17A and/or IL-17F plays a role in regulating neutrophilic and macrophage inflammation, modulating activation of the lower airway structural cells in COPD and may drive autoimmune responses [34, 35]. IL-17 is also expressed by other cells including IL-17-producing γδ T (γδ T-17) cells, natural killer T-17 cells and IL-17-producing lymphoid tissue-induced cells [36]. The expression of both IL-17A and IL-17F is increased by cigarette smoke exposure in lung explants from both non-COPD and COPD subjects, supporting that local lung cells contribute IL-17 production. Finally, sputum IL-17A levels are increased in stable COPD patients compared with control subjects [37]. Elevated IL-17A/F expression is dependent on NF-κB and PI3K pathways [38].

The number of IL-17A+ and IL-22+ immunoreactive cells is increased in the bronchial submucosa of stable COPD compared with control nonsmokers [39] although the expression of IL-17F and IL-21 is not significant different among subject groups [39]. IL-17A and IL-17F staining was observed in endothelial cells and in inflammatory cells and fibroblasts [39]. Interestingly, in this study, we observed that <5 % of IL-17+ cells were T cells and >90 % of IL-17A+ cells were CD31+ endothelial cells. Furthermore, in IL-22, <10 % of IL-22+ cells were T cells and >80 % of IL-22+ cells were CD31+ endothelial cells [39].

In addition, the number of IL-22+ and IL-23+ immunoreactive cells is increased in the bronchial epithelium of stable COPD compared with control groups. In all smokers, with and without disease, and in patients with COPD alone, the number of IL-22+ cells correlated significantly with the number of both CD4+ and CD8+ cells in the bronchial mucosa. RORC2 messenger RNA (mRNA) expression in the bronchial mucosa was not significantly different between smokers with normal lung function and COPD [39].

The number of inflammatory cells expressing IL-17A in the small airway subepithelium is higher in patients with COPD than in control. IL-17A was expressed by lymphocytes, neutrophils and macrophages [40]. The expression of IL-17F is greater than IL-17A in epithelial cells and lymphoid follicles but without significant differences among subject groups, whereas IL-17A expression is higher than IL-17F in the subepithelium [40].

IL-17A expression is significantly elevated in severe to very severe stable COPD (GOLD III/IV) compared with both smokers and never smokers without COPD. Although CD3+ T cells express IL-17A in very severe COPD, most IL-17A+ cells are tryptase-positive mast cells [41]. CXCL12 is highly expressed in lymphoid follicles in COPD lungs, and the pulmonary expression was significantly elevated in end-stage COPD [41]. This suggests that IL-17A in the peripheral lung of patients with severe to very severe COPD may contribute to disease progression and development of lymphoid follicles via activation of CXCL12 [41].

In contrast, the COPD patients had significantly lower relative and absolute numbers of γδ T cells in induced sputum and in BAL compared with those from healthy nonsmoking subjects as well as of blood Th10 cells [42]. The quantity of γδ T cells negatively correlated with FEV1 and pack-years of smoking only in COPD group [43].

Aberrant lung CD4+ T cells polarisation do not only appear to be common in advanced COPD but also exists in some smokers with normal lung function and may contribute to development and progression of specific COPD phenotypes [44].

Analysis of unstimulated lung CD4+ T cells of all subjects identified a molecular phenotype, mainly in COPD, characterised by markedly reduced mRNA transcripts for transcription factors controlling Th1, Th2, Th17 and FOXP3+ T regulatory subsets and their signature cytokines. As a group, these subjects had significantly worse spirometry but not DLCO. Unbiased analysis of unstimulated lung CD4+ T cell signatures identified two distinct molecular phenotypes which correlated with clinical features: IL-10 expression correlated independently and inversely with emphysema but not with spirometry and IFN-γ expression correlated independently and inversely with reduced spirometry but not with reduced DLCO or emphysema [44]. Stimulation of COPD cells induced minimal IFN-γ or other inflammatory mediators, although many patients produced more CCL2, and the T effector memory subset was less uniformly predominant and did not correlate with decreased IFN-γ production [44].

### T regulatory lymphocytes in the COPD immunopathology of stable patients

A deficiency in CD4+CD25+FOXP3 regulatory T cells (Tregs) can impair the immune system’s tolerance for autoantigens and thereby lead to immune disease [45, 46]. Tregs represent between 1 and 3 % of total CD4+ T cells and accumulate at tissue sites of antigen invasion where they exert site-localised immune suppression by producing IL-10 and transforming growth factor (TGF)-β1. The intracellular expression of FOXP3 is currently considered as the most specific marker for human Treg cells [47].

In patients with stable COPD there is decreased blood number of CD25++ CD45RA+ resting Tregs (rTregs) and CD25+++CD45RA- activated Tregs (aTregs), which are suppressive, and a significantly increased number CD25++CD45RA-cytokine-secreting (Fr III) Tregs compared with control smokers with normal lung function [48]. In addition, Tregs from patients with stable COPD suppress T cell proliferation to a greater extent than Tregs from healthy subjects contributing to effector T cell dysfunction in COPD [49]. Sputum FOXP3 mRNA levels are decreased in stable COPD patients compared with control smokers with normal lung function [50] and Treg numbers are decreased in the BAL of stable COPD compared with control smokers with normal lung function [31].

There is debate over Treg numbers in COPD tissue. The number of CD4+CD25+FOXP3 Tregs in the bronchial biopsies [51] or lungs [52] of patients with stable COPD is not significantly different compared with healthy controls but is decreased in the small airways of COPD patients, and this negatively correlates with the degree of airflow obstruction [47]. Another study has also demonstrated decreased CD4+CD25+ Treg cell number in lung tissue from emphysema patients, which in turn correlated with FOXP3 mRNA expression [53].

### B lymphocytes, lymphoid follicles, autoimmunity and COPD immunopathology in stable patients

**Fig. 5:**
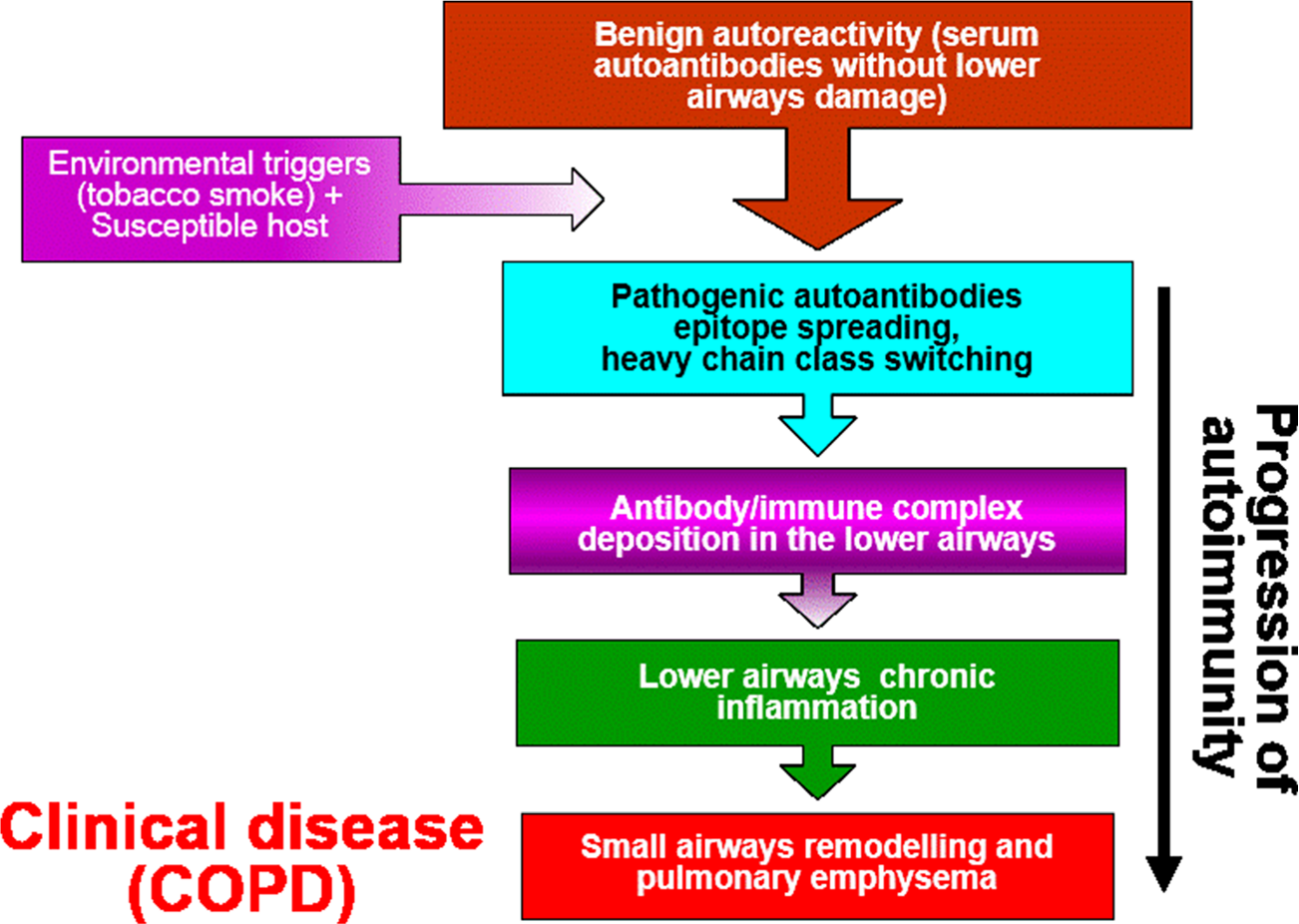
Autoimmunity develops in COPD when environmental triggers transform the production of benign autoantibodies to pathogenic antibodies in susceptible subjects. Small airways remodelling and emphysema develops with the deposition of immune complexes within the alveoli

There is mounting evidence that autoimmunity has a role in the pathogenesis of COPD [45, 46]. The role of B lymphocytes in the pathogenesis of stable COPD is unknown, but they may secrete autoantibodies directed against oxidised extracellular matrix proteins or against endothelial cells (Fig. 5). Carbonyl-modified proteins, arising as a result of oxidative stress, promote auto-antibody production [54]. The serum antibody titer against carbonyl-modified self-protein significantly increases in patients with severe COPD compared with control subjects. Antibody levels correlate inversely with disease severity and are predominantly IgG1 in type. Deposition of activated complement in the vessels of peripheral COPD lung as well as autoantibodies against endothelial cells is also seen [54].

An infiltration of B lymphocytes into the adventitia of the small airways of COPD patients has been reported and the percentage of the small airways that contain B cells and lymphoid aggregates containing well-demarcated follicles with germinal centres increases as the severity of COPD progresses [8, 11]. These lymphoid collections are rarely observed in the small airways of nonsmokers, are present in the small airways of ∼5 % of smokers with normal lung function, as well as in smokers with mild to moderate COPD and increasing to 25–30 % of airways in severe and very severe COPD [8, 11]. A more recent study has observed an increased number of B cells in the connective tissue (but not in the epithelium or smooth muscle) only in the small airways of patients with very severe COPD compared with the other control group of subjects [55].

The lymphoid follicles in the small airways of COPD patients are composed of large aggregates of B lymphocytes with interspersed CD21+ and CD35+ follicular dendritic cells [56] surrounded by lower numbers of CD4+ (80–90 %) and few CD8+ T lymphocytes [8, 11, 57]. The B lymphocytes are mainly IgM-bearing but IgD negative, which suggests that they may have been activated to some extent. Moreover, a predominant part of the infiltrate is CD27+, a marker for memory B lymphocytes [56].

The B lymphocytes are oligoclonal [57] suggesting that they play a role in local antigen-specific immune responses. It is not known whether microbial antigens, cigarette smoke-derived antigens or antigens from extracellular matrix breakdown products are important or if this response is beneficial or detrimental [56].

The presence of B cell-activating factor belonging to the tumour necrosis factor (TNF) family (BAFF) is increased within follicles in severe and very severe COPD patients [58, 59], around CD4+ cells, dendritic cells, follicular dendritic cells and fibroblastic reticular cells [60] as well as in patients with emphysema [61]. BAFF is responsible for B cell survival and maturation and is a Th1 response-promoting cytokine [58].

In the National Emphysema Treatment Trial (NETT) study, there was a strong trend toward a reduction in the number of airways containing lymphoid follicles in severe and very severe COPD patients receiving oral and/or inhaled glucocorticoids [62]. This may account for the increased risk of pneumonias in patients on high-dose inhaled glucocorticoids observed in long-term controlled clinical trials, such as the TORCH study [63].

CD57+ expression in T lymphocytes is a marker of in vitro replicative senescence [64]. The density of CD57^+^ cells within lymphoid follicles of COPD patients is significantly increased compared with nonsmokers and smokers without COPD. In support of this, telomere-associated DNA damage foci increased in small-airway epithelial cells from patients with COPD without significant telomere shortening detected [65]. Moreover, the percentage of lymphoid follicles with cell apoptosis is also significantly higher in COPD patients supporting the hypothesis of a local immune dysfunction in COPD [66].

### Macrophages and COPD immunopathology in stable patients

CD68+ macrophages are increased in the bronchial mucosa of mild/to moderate stable COPD patients compared with control subjects [15, 17, 18]. There was originally some controversy as to the expression of CD68+ cells in the small airways, but it is now evident that the number of macrophages in the small airways increases as the severity of COPD progresses compared with control smokers with normal lung function [8, 11].

Macrophages may play an important role in orchestrating the inflammation in COPD lungs through their release of multiple pro-inflammatory mediators including proteases, such as matrix metalloprotease-12, cytokines, chemokines and oxidants [15–18] and also have a reduced phagocytic ability which may drive the persistence of inflammation and impair the clearance of infectious pathogens and apoptotic cells [35].

Macrophages constitute a heterogeneous cell population, and there is no clear evidence for a predominance of classical M1 or M2 macrophages in COPD, and an intermediate phenotype may be present [67] including a glucocorticoid insensitive phenotype [68]. Indeed, using transcriptomic signatures quite distinct from macrophage phenotypes have been associated with smoking, which are absent in COPD [69] suggesting that the surrounding pulmonary environment in COPD may generate a specific phenotype that is permanently altered compared with that seen in smokers. Other studies show that the numbers and percentages of CD163+, CD204+ or CD206+ alveolar macrophages in patients with COPD at GOLD stages III and IV are significantly higher than in those at GOLD stages I and II and those in smokers and nonsmokers with a significant negative correlation between the number of CD163+, CD204+ or CD206+ alveolar macrophages and the predicted FEV1 [70].

### Dendritic cells and COPD immunopathology in stable patients

The number and the functional subsets of dendritic cells (DCs) in the lower airways of COPD patients compared with control subjects is still controversial. This is an area of active research in COPD immunopathology [71]. Mature CD83+ DCs are decreased in sputum of stable COPD patients compared with never smokers and smokers with normal lung function after smoking cessation [72]. Using transmission electron microscopy (TEM), DC numbers were significantly decreased both in the epithelium and subepithelium of current smokers with COPD compared with ex-smokers with COPD and healthy controls [73]. Furthermore, patients with moderate/severe stable COPD had significantly fewer mature CD83+ DCs and an increased CD207/CD83 DC ratio in their bronchial mucosa compared with nonsmoking subjects [74].

BAL mDCs of current smokers, but not ex-smokers, with stable COPD have an increased expression of receptors for antigen recognition such as CD1c or Langerin but reduced CD83 expression, as compared with never-smoking controls status [75]. The chemokine receptor CCR5 on myeloid DCs, which is important for the uptake and procession of microbial antigens, is strongly reduced in all patients with stable COPD, independently of the smoking status [75].

The volume density (i.e. the volume of DCs as the percentage volume of the airway wall) comprising CD83+ (mature) DCs is also significantly reduced in the small airways of patients with stable COPD vs smokers with normal lung function and never smokers [76]. In contrast, a more recent study found reduced numbers of CD83+ and CCR7+ DCs and an increased number of CD1a+ DCs in the small airways of patients with stable COPD vs smokers with normal lung function [77] although other studies show no increase in CD1a+ DCs was found [78] and an increase in the total number of CD83+ DCs in the peripheral lung of patients with stable COPD vs smokers with normal lung function [79]. Overall, it is likely that cigarette smoke may stimulate immune responses by impairing the homing of airway immature DCs to the lymph nodes and reduce the migratory potential of immature DCs [77].

A higher number of Langerhans-type DCs (LDCs) was reported in the small airways of current smokers without COPD and in COPD patients compared with never smokers and ex-smokers without COPD, but there was no difference in the number of LDCs between current and ex-smoking COPD patients. In contrast, the number of interstitial-type DCs (intDCs) did not differ between study groups [80]. Interestingly, the number of CD1c+ DCs is significantly decreased in the lower airways of stable COPD patients compared with never smokers and further decrease with the severity of the disease.

### Neutrophil granulocytes and COPD immunopathology in stable patients

Neutrophils accumulate in the sputum and BAL of stable COPD patients [15, 17, 18, 81, 82] in response to the increased expression of macrophage inflammatory protein-1 (MIP-1α) in the bronchial epithelium of severe stable COPD patients in comparison with subjects with mild/moderate disease and control smokers [15, 17, 18]. Increased myeloperoxidase (MPO) immunoreactivity in comparison with mild/moderate disease and both control groups is also seen [15, 17, 18]. In the submucosa, we reported a further increase of neutrophils and macrophages (CD68+) in comparison with control smokers [15, 17, 18] and decreased numbers of T lymphocytes (CD3+ cells) and of CD3+ cells co-expressing the CCR5 receptor (CCR5+CD3+ cells) in comparison with both mild/moderate COPD patients and control smokers [28].

We have also shown increased MPO+ and nitrotyrosine+ (NT) cells in the submucosa of severely diseased patients in comparison with mild/moderate COPD, control smokers and nonsmokers [15, 17, 18]. Nitrotyrosine formation is related to peroxynitrite activity, which causes tissue damage, the release of small pro-inflammatory peptides and increased adhesion and activation of tissue neutrophils and macrophages. Since IL-8 induces MPO release from neutrophils this may drive a feedforward process to sustain inflammation.

Interestingly, our analysis of pro-neutrophilic chemokines showed higher levels of CCL5 (RANTES) in epithelium and increased numbers of CCL5+ and CXCL7+ (NAP-2) cells in the submucosa of severe stable COPD patients when compared with control nonsmokers [15, 17, 18]. We also found an increased neutrophilic expression of the extracellular matrix components CD44 and CD11b in the bronchial mucosa of severe COPD compared with control smokers [15, 17, 18], suggesting a role particularly for CC chemokines (RANTES, MIP-1α) in substaining neutrophilia in patients with severe disease. Increased adhesiveness of neutrophils may also contribute to increased permanence of these cells in the bronchial tissue of severely diseased patients [15, 17, 18] (Fig. 2).

Increased neutrophil numbers are found in the small airways as the severity of COPD progresses compared with control smokers with normal lung function [8]. These data together show a shift of the cellular types involved in severe stable COPD, with a prevalence of cells possessing phagocytic and proteolytic activity in the bronchial tissue. In contrast, the T cell-mediated immunoresponse could be impaired or modified in COPD patients with severe disease [28].

In contrast to asthma, most studies show no significant differences in the number of mast cells (tryptase+) or eosinophil granulocytes identified with histochemical staining or as EG2+ cells in the wall of the small airways of COPD patients compared with control smokers with normal lung function [8, 23, 83, 84].

## Structural cells of the wall of the lower airways in COPD immunopathology in stable COPD patients

### Lower epithelium airways in COPD immunopathology in stable COPD patients

For COPD to develop, cigarette smoke has to bypass or overwhelm the host front lines of defence, namely the respiratory tract mucosal epithelium, which serves as an effective physical barrier and the innate immune system, which provides an immediate, yet nonspecific response [85]. Lower airways epithelial cells (AEC) and DC are situated in close proximity within the airway epithelium and are the first cells to encounter inhaled pathogens and environmental pollutants/irritants. AEC and DC interact through the release of cytokines, chemokines, proteases and other soluble mediators and through direct cell-cell contact, and these interactions are likely to play an important role in maintaining immune homeostasis [86].

At variance with asthma, the functional activity of bronchial epithelium in COPD patients is poorly studied and most studies have focused on the role of mucus-secreting epithelial cells and on epithelial stem cells.

NF-κB is activated in the bronchial epithelium of mild/moderate stable COPD patients and, to a lesser extent, in control smokers in comparison with control nonsmokers [27]. NF-κB activation may account for the increased expression of pro-inflammatory cytokines such as IL-1, IL-6, IL-8, MCP-1, TNF-α and ICAM-1 in COPD [27]. For example, the expression of the adhesion molecule ICAM-1 is increased in the bronchial epithelium in mild/moderate stable COPD in comparison with both control nonsmokers and current smokers [15, 87] and may play a role in the T cell epithelial adhesion [15] and T cell-mediated response to viral infections [15, 88].

### Mucus-secreting epithelial cells and intraluminal mucus in COPD immunopathology

The pathophysiological relationship between airway mucus secretion and COPD are complex. Many patients with COPD have chronic bronchitis with increased sputum production. The presence of chronic bronchitis is a predictor of COPD-related death, increased risk of pneumonia and of an accelerated decline in lung function [89, 90]. Mucins are the main component of lower airway mucus, and several mucins (including MUC2, MUC5AC, MUC5B, MUC6 and MUC8) are secreted in the lower airways [91–94]. The occlusion of the small airways by inflammatory exudates containing mucus was associated with early death in patients with severe emphysema [62].

There are increased amounts of MUC5B and MUC5AC in the sputum of stable COPD patients with little MUC2 [95–97] but no difference in bronchial submucosal gland size [91]. MUC5AC expression is increased in the bronchial surface epithelium both in smokers with normal lung function and with COPD compared with the control group of nonsmokers [91]. This confirms and extends the results of previous studies that have found increased expression of MUC5AC in bronchial surface epithelium from smokers (with or without COPD) [98, 99]. In addition, there is a significant correlation between MUC5AC expression and pack-years [91], highlighting the potential of tobacco smoking (and its components such as acrolein and oxidants) to activate MUC5AC in bronchial epithelial cells via NF-κB [94]. In contrast, MUC5AC expression is elevated in bronchial submucosal glands of COPD patients compared with both smokers with normal lung function and nonsmokers (control groups) [91]. The expression of MUC5B in both bronchial surface epithelium and submucosal glands was not significantly different between groups.

In the NETT, when the pathological changes of the small airways in the resected lung specimens were correlated with clinical outcomes after surgery, the investigators found that occlusion of the small airways by inflammatory exudates containing mucus was associated with early death in patients with severe emphysema [62]. Interestingly, in excised lungs obtained from patients with severe emphysema, the introduction of a catheter to the small airways and their irrigation with a mucolytic drug reduces significantly the peripheral lung resistance [100].

The number of PAS+ or Alcian Blue+ goblet cells is not significantly different in the small airways of patients with mild to moderate stable COPD compared with control subjects with normal lung function [23, 101]. In contrast, there is an increase of intraluminal PAS+ (neutral mucins) and MUC5B+ mucus and of the expression of MUC5AC in the bronchiolar epithelium of the small airways in patients with COPD compared with smokers with normal lung function. These changes may contribute to the pathogenesis of the small airways obstruction and of the increased risk of pneumonia of the COPD patients [101].

### Nerves, neuromediators/neurotransmitters, neuroendocrine cells, neuropeptides, neurogenic inflammation and COPD immunopathology

The cross-talk between the immune system and the neuroendocrine system of the lower airways is complex and scarcely investigated in patients with COPD. It is now evident that immune cells release many cytokines, chemokines, neuromediators/neurotransmitters and neuropeptides, which, in turn signal to the central and peripheral nervous system and the immune system [102] and in vitro ACh might promote Th17-differentation [103].

Pilot data suggest that 5-HT and CGRP receptor distribution is altered in the lung of patients with stable COPD compared with controls. The 5-hydroxytryptamine receptor 1F (HTR1F) is expressed in nearly all basal cells in COPD compared with only a subset of basal cells in control subjects [104]. In contrast, there is decreased expression of the CGRP receptor, calcitonin receptor (CALCR), in the airway epithelium in patients with COPD, with increased localization to basal cells in the control subjects. Similarly, HTR2B has a diffuse epithelial expression in COPD with an increased basal cell staining in controls [104].

In the central airways of patients with stable COPD, compared with control smokers with normal lung function, there is an increased immunoreactivity for substance P (SP) and VIP, paralleled by a decreased NPY expression in the epithelium and glands, and a decreased expression of all these three neuropeptides in the smooth muscle layer [105]. In addition, the density of VIP-positive nerves is significantly increased in the bronchial submucosal glands of smokers with chronic bronchitis than in nonbronchitic subjects [106]. In conjunction, there is increased expression of the VIP receptor VPAC1R, but not VPAC2R, in the bronchial epithelium, bronchial glands and vessels of smokers with symptoms of chronic bronchitis compared with asymptomatic smokers with normal lung function. These subjects also had an increased number of mononuclear cells positive for both VPAC1R and VPAC2R in the bronchial submucosa [107]. The functional role of these abnormalities in neuropeptide pathways present in the lower airways of patients with COPD is unknown.

### Endothelial cells of the lower airways in COPD immunopathology

The expression of the adhesion molecule ELAM-1 is increased in the bronchial mucosa endothelium in mild/moderate stable COPD patients in comparison with both control nonsmokers and current smokers [15, 17, 18, 87] and may be involved in the neutrophil recruitment in COPD.

We investigated the immunoexpression of the Th17 related cytokines in the bronchial biopsies of patients with increasing COPD severity and observed significantly higher levels of epithelial IL-22 and submucosal IL-22 and IL-17 in patients with mild/moderate COPD compared with control nonsmokers, suggesting a role for these Th17 related cytokines in substaining tissutal neutrophilia in the bronchi of these patients [39].

Interestingly, in that study, we observed that less than 5 % of IL-17+ cells were T cells and more than 90 % of IL-17A+ cells were also CD31+ endothelial cells. Considering IL-22, less than 10 % of IL-22+ cells were T cells and more than 80 % of IL-22+ cells were also CD31+ endothelial cells, suggesting that the contribution of T cells in producing the IL-17-related cytokines and their possible related neutrophilic effects is relatively modest and that surprisingly, endothelial cells contribute importantly to the total upregulation of these cytokines in the bronchial mucosa of COPD patients [39]. Furthermore, upregulation of these pro-neutrophilic cytokines starting at milder stage of the disease indicates a possible differential role with pro-neutrophilic chemokines (CCL5, CXCL7) becoming prevalent at a more severe stage of the disease [15, 17, 18].

In some studies, there is an increase in apoptotic alveolar epithelial and endothelial cells in the peripheral lungs of severe stable COPD patients with severe pulmonary emphysema [108, 109]. If this is not counterbalanced by an increase in proliferation of these structural cells, the net result is destruction of lung tissue and the development of pulmonary emphysema [110].

Vascular endothelial growth factor (VEGF) expression is significantly decreased in the bronchiolar epithelium of smokers with COPD compared with smokers with normal lung function. However, bronchiolar VEGFR-2 is downregulated in smokers with and without COPD in comparison to lifelong nonsmokers only [111]. However VEGF, VEGFR-2, GYP-1 and NRP-1 expression in the peripheral lungs of patients with stable COPD is not reduced compared with control smokers with normal lung function [112] and VEGF expression is increased in the wall of small pulmonary arteries of patients with stable COPD compared with control smokers with normal lung function [113].

Endothelial dysfunction of the small pulmonary arteries, which is associated with decreased release of endothelium-derived vasodilating agents (nitric oxide, prostacyclin) and increased expression of growth factors and vasoconstrictive agents (such as endothelin-1) is present in all patients with stable COPD from mild to severe. In these patients, as well as in smokers with normal lung function, the small pulmonary arteries show thickened intimas with CD8+ T lymphocyte infiltration [114–116].

Pulmonary hypertension is a common complication in COPD. Its presence and severity is closely related to disease prognosis. Remodelling of pulmonary vessels is the principal causative factor of pulmonary hypertension in COPD [114–116]. In advanced COPD, pulmonary vascular remodelling is related to the severity of arterial hypoxaemia. However, structural abnormalities and alterations of vascular function are also apparent in patients with mild COPD who do not have hypoxaemia and even in smokers with normal lung function [114–116].

### Drugs effect on COPD immunopathology

In contrast to asthma, glucocorticoid treatment of stable COPD is rather ineffective in reducing airway inflammation and the decline of lung function [15]. However, inhaled glucocorticoids (improperly termed inhaled corticosteroids (ICS)) such as budesonide and fluticasone, delivered alone or in combination with a LABA, are associated with increased risk of serious adverse pneumonia events, but neither significantly affected mortality compared with controls [117, 118]. This suggests that ICS may alter immune responses in the COPD lungs increasing their susceptibility to bacterial infections.

Few data are available in the literature regarding changes in the immunopathology after treatment with inhaled glucocorticoids in the bronchial biopsies and/or bronchoalveolar lavage of stable COPD patients. A recent systematic review [119] showed that ICS were effective in reducing CD4 and CD8 cell counts in bronchial biopsies and reduced BAL neutrophil and lymphocyte counts but increased BAL macrophage counts. Some studies also observed a significant decrease in BAL inflammatory mediators such as CXCL8 [120] and PGE2, 6kPGF1alpha and PGF2alpha [121]. Data obtained from the sputum analysis [122, 123] are in agreement with the few observations reported for bronchial biopsies and BAL.

Three months salmeterol/fluticasone propionate reduced CD8+ and CD68+ cells in bronchial biopsies of moderate/severe COPD compared with placebo-treated subjects [124] whereas 6 to 30 months treatment with or without added bronchodilators showed decreased counts of CD3+, CD4+ and mast cells in moderate/severe COPD patients compared with the placebo group [125]. In addition, the combination of inhaled fluticasone propionate plus salmeterol, but not fluticasone propionate alone, reduced the number of BAL myeloid DCs, macrophages and neutrophils in current smokers with normal lung function compared with placebo [126].

### Inflammatory mediators and oxidative stress in the pathogenesis of smoking-induced COPD

COPD is characterised by enhanced oxidative stress and expression of many pro-inflammatory proteins including cytokines, chemokines, growth factors, neuropeptides, enzymes, receptors and adhesion molecules and reduced expression of anti-inflammatory mediators, such as IL-10, in the lower airways and lungs [7, 10, 15, 127–131].

Both reactive oxygen species (ROS) and reactive nitrogen species (RNS) play a role in the pathogenesis of smoking-induced COPD [131–134]. The respiratory system is chronically exposed to environmental pollutants, including oxidants, and the inhaled gas component of cigarette smoke may contain as many as 10^14–16^ free radicals per puff. Free radicals are highly reactive, carbon- and nitrogen-centred species. Although generally short-lived (<1 s), the half-life of many of these is sufficient to reach the lower respiratory tract. As much as 500 ppm of nitric oxide (NO) exists in cigarette smoke, and cigarette smoke converts tyrosine to 3-nitrotyrosine and dityrosine [131–134]. In addition, COPD inflammatory cells have a heightened capacity to produce oxidants. Activated neutrophils, macrophages and resident cells such as epithelial cells and airway smooth muscle cells can generate oxidants particularly after pro-inflammatory mediator stimulation [129, 130]. RNS are mainly produced by reaction between and nitric oxide (NO) and O_2_− yielding the powerful radical peroxynitrite (ONOO−), the production of which is increased in the lower airways of patients with COPD and may contribute to mucus hypersecretion and lower airways damage [135].

**Fig. 6:**
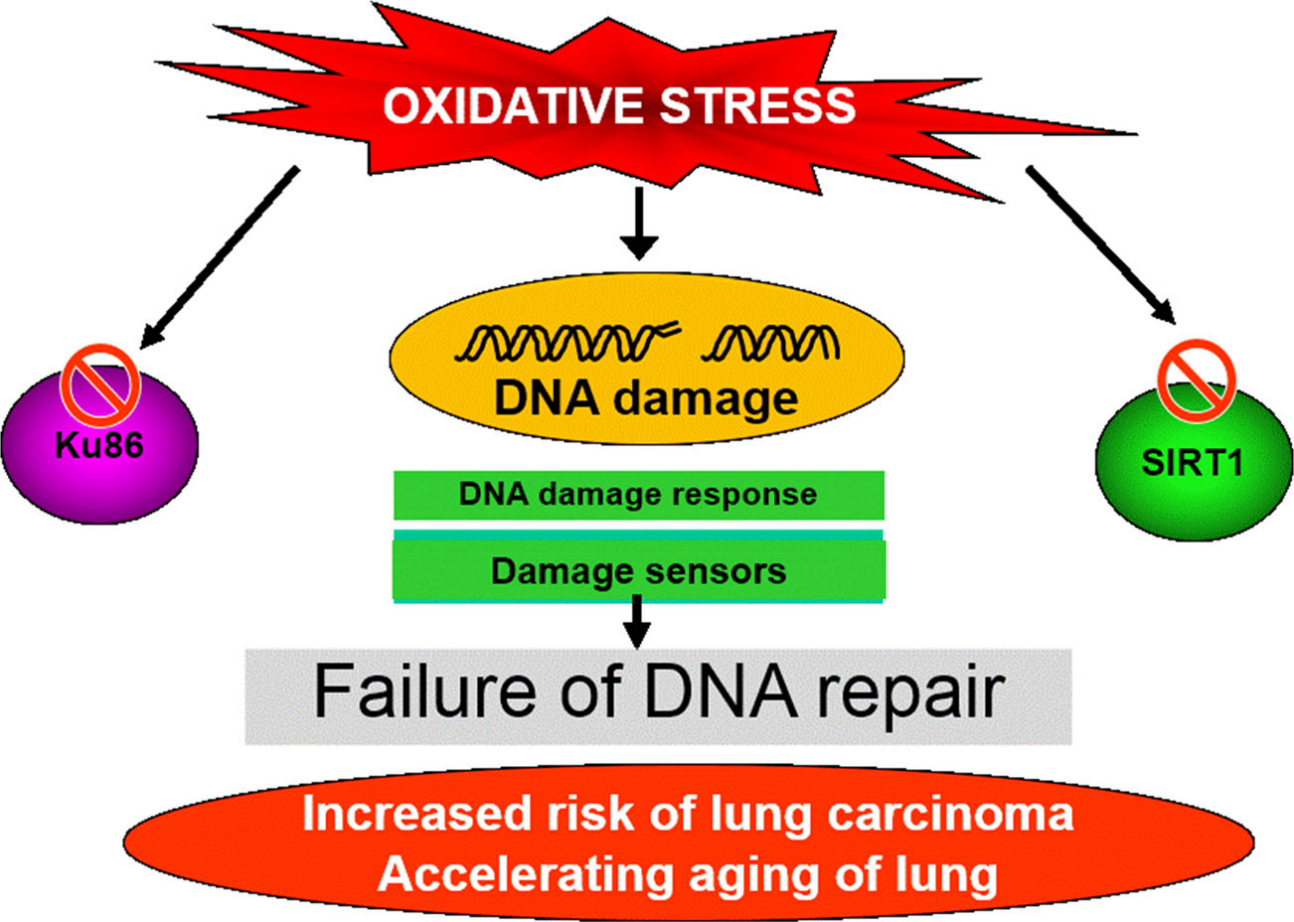
The increased risk of lung cancer seen in COPD patients may result from abnormal DNA damage/repair processes in response to oxidative stress. Cigarette smoke-induced oxidative stress causes DNA strand breaks and a reduction in the expression of key repair protens such as Ku86 and sirtuin 1 (*SIRT1*). The resultant failure to repair the damaged DNA foci leads to accelerated lung ageing and cancer

Oxidants participate in many signal transduction pathways targeting cell growth and proliferation, as well as homeostatic mechanisms. For example oxidant stress enhances the disruption of mitochondrial transport chain [136] leading to either cell injury (necrosis) or apoptosis. Carbonyl stress, a form of oxidative stress causes nonenzymatic posttranslational modifications on proteins that alter protein function resulting in the formation of danger-associated molecular patterns (DAMPs) and neo-autoantigens (Fig. 5). Carbonyl stress-induced damage to mitochondrial proteins drives further ROS production by the damaged mitochondria. Thus, carbonyl-modified proteins help drive the autoimmune mechanisms in COPD [131]. Furthermore, smokers with normal lung function and patients with stable COPD show increased oxidative DNA damage (8-hydroxyguanine formation) in their lungs as compared with nonsmokers and this may contribute to the increased risk of lung cancer observed in the patients with stable COPD [17] (Fig. 6).

Only a fraction of lifelong smokers develop COPD [137]. It is possible that oxidative stress may overcome antioxidant defences in the lower-airway epithelium in susceptible subjects and thereby promote COPD pathogenesis [131–134]. These data suggest that the inhibition of intracellular oxidative stress may be a potential therapeutic target for treatment of virus-induced COPD exacerbations.

### Cytokines and chemokines in COPD immunopathology

There is a heightened inflammatory response in the lower airways and lungs of patients with stable COPD associated with enhanced expression of a number of key cytokines including TNF-α, IFN-γ, IL-1β, IL-6, IL-17, IL-18, IL-32 and TSLP and growth factors, such as TGF-β [22, 39, 130].

**Fig. 7:**
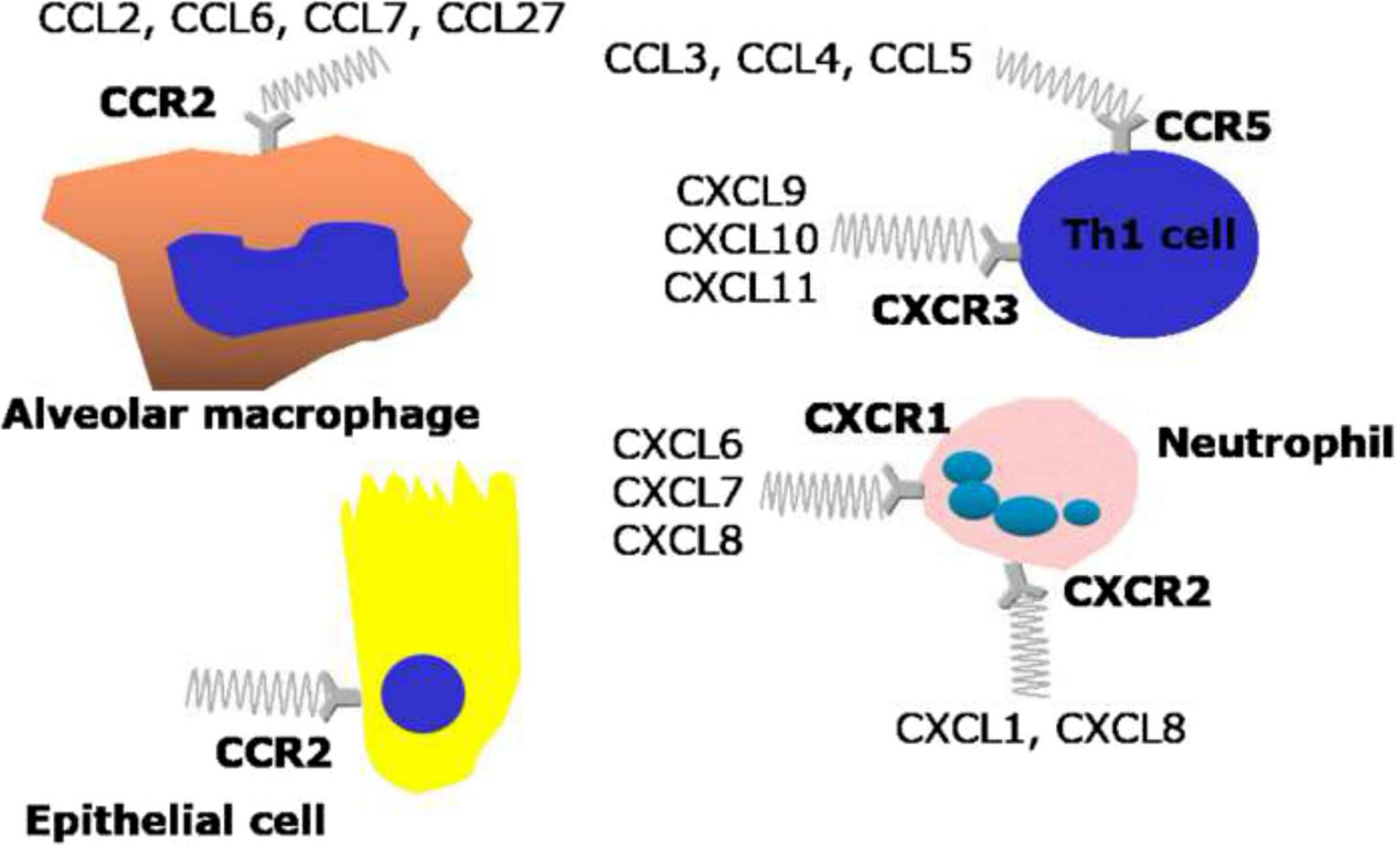
Key chemokines such as CCL2, CCL3, CCL4, CCL5, CCL6, CCL7 and CCL27 and CXCL6, CXCL7, CXCL8, CXCL9, CXCL20 and CXCL11 in COPD mediate their effects through targeting specific chemokine receptors including CCR2, CCR5, CXCR1, CXCR2 and CXCR3 localised on alveolar macrophages, Th1 cells, neutrophils and airway epithelial cells

Many studies have described increased/decreased expression of selected chemokines and/or chemokine receptors (summarised in Fig. 7) in different compartments of the lower airways in patients with COPD. It is imperative to better understand the chemokine-related inflammatory mechanisms that underlie inflammatory cell recruitment into COPD airways using primary cells and tissues from COPD patients to design better therapeutics. Compounds that target chemokines and their receptors are still in the early stages of development, and the results of phase II clinical trials are awaited with great interest. The potential for using chemokines as biomarkers to predict clinical phenotypes or progression of COPD or even to identify responders to particular therapies has so far not been realised. It may be the case that certain clinical phenotypes respond better to specific therapies [129].

### Immunopathology of COPD exacerbations

**Fig. 8:**
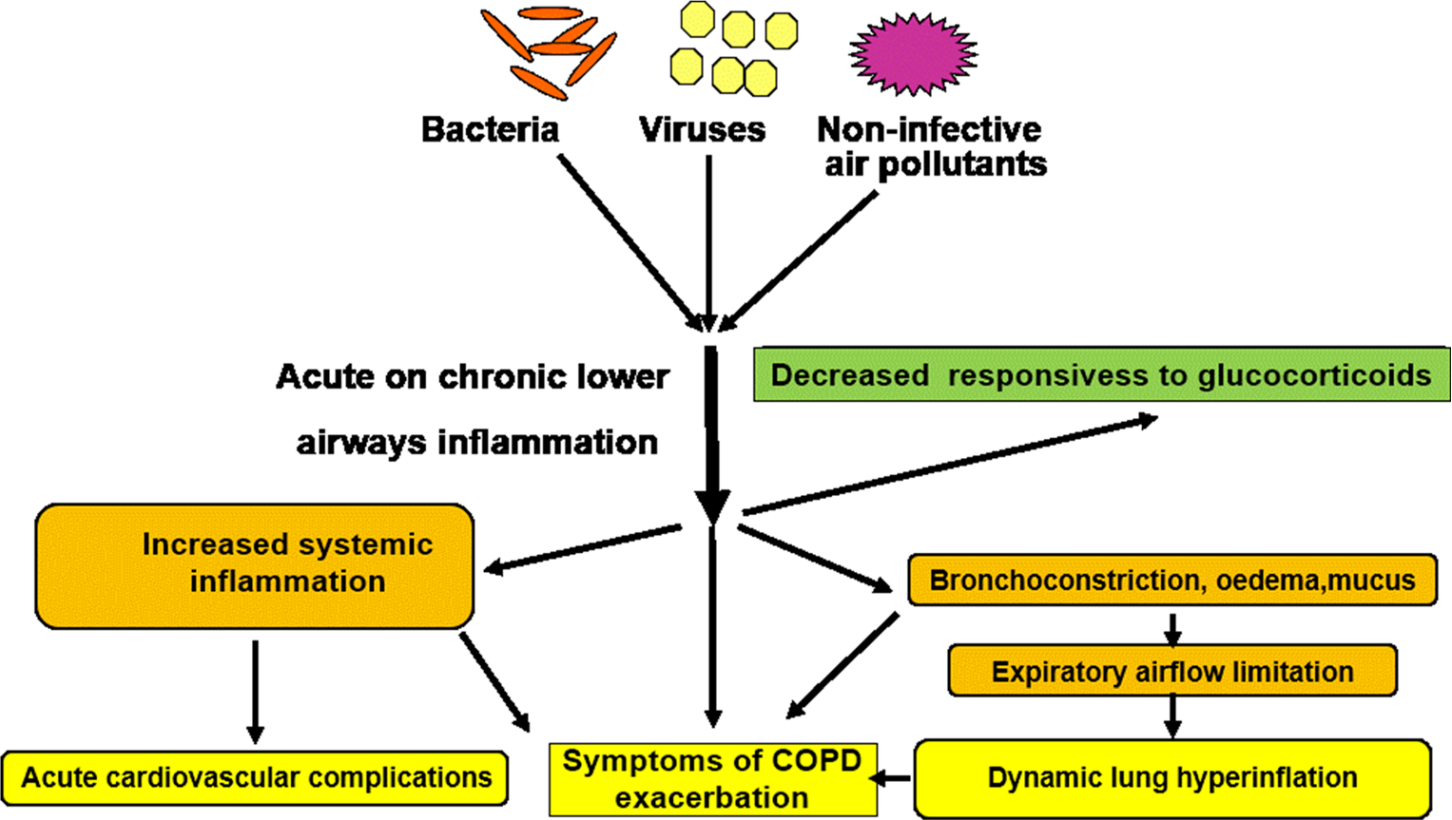
The presence of viruses, bacteria and air pollutants can drive COPD exacerbations by causing an acute-on-chronic inflammatory state within the airways. This results in systemic inflammation causing cardiovascular complications, airway bronchoconstriction oedema, mucus and lung hyperinflation which together are associated with the signs and symptoms of an exacerbation. This acute-on-chronic inflammation is also associated with reduced responsiveness to inhaled and systemic glucocorticoids

There is an increased number of sputum neutrophils during severe COPD exacerbations leading to acute respiratory failure that is independent of the type (bacterial or virual) of infectious agent detected (Fig. 8); whereas, virus-induced COPD exacerbations with or without concomitant bacterial infection are associated with an increased number of sputum eosinophils, suggesting that sputum eosinophilia could be a marker of viral infection during COPD exacerbations [132, 133]. Interestingly, increased sputum CD8+ T lymphocytes have been reported during COPD exacerbations with a relative reduction in the ratio of IFN-γ/IL-4 expressing CD8+ T lymphocyte [138, 139]. Thus, a switch towards a Tc2-like immunophenotype during COPD exacerbations could trigger recruitment of eosinophils, a classical effector cell recruited during Tc2 mediated immune responses, during virus-induced COPD exacerbations.

Patients with mild/moderate COPD exacerbations show an increased number of eosinophils in their bronchial mucosa and increased mRNA for CCL5 (RANTES), neutrophils, T lymphocytes, very late antigen (VLA)-1 and TNF-α in comparison with stable COPD patients [15, 17, 18] (Table 1). Increased eotaxin-1 and CCR3 chemokine receptor expression has also been reported [15, 17, 18]. Severe exacerbations of COPD were associated with increased neutrophilia and upregulation of epithelial mRNA for CXCL-5 (ENA-78), CXCL-8 (IL-8), CXCR-1 and CXCR-2 in comparison with stable disease [140]. Thus, total inflammation, involving different inflammatory cells, is significantly increased during COPD exacerbations. However, the eosinophilia observed during exacerbations in mild/moderate COPD, differs from that reported in asthma since the presence of eosinophils is transitory and frequently confined to capillary vessels [15, 17, 18].

**Table 1:** Variations in inflammatory cells and related markers in the bronchial submucosa during COPD exacerbations

	Cell type			Inflammatory mediator					
Upregulated	Eosinophils	Neutrophils	CD3 T cells	VLA1	TNF-α	CCL5	CXCL5	CXCL8	CXCR1
Unchanged	Mast cells	CD68 macrophages		ELAM1	ICAM1	IL-4	IL-5		

Data extracted from ([33, 138–140] and references therein)

Not surprisingly, almost all of the studies on immunopathology of COPD in the small airways have been conducted on specimens obtained during stable conditions. The largest study on the pathology of the small airways during COPD exacerbations has showed that death from COPD is associated with both emphysema and small airway inflammation [141, 142].

### In vivo model of RV-induced COPD exacerbation

A human experimental model of infection in COPD patients allows studies to take place under controlled conditions [143, 144]. This model showed that experimental rhinovirus infection can cause exacerbation in COPD patients. COPD patients developed colds and exacerbations with 100- to 1000-fold lower doses of virus than used in previous studies in asthmatic and normal volunteers, and there was a 3- to 4-day gap between the peak of cold symptoms and the peak of lower respiratory symptoms [143, 144]. Interestingly, at variance with smokers with normal lung function, sputum neutrophils significantly increased in COPD patients following experimental infection. BAL CD3+ and CD8+ T cells increased in COPD patients post-infection compared with baseline and CD3+ T cells correlated with virus load [143, 144]. BAL IL-27 and IL-10 levels are also increased [143, 144]. There were also significant increases in airway inflammation and markers of oxidative and nitrosative stress in COPD subjects which correlated with virus load. Sputum macrophage HDAC2 activity pre-infection was inversely associated with sputum virus load and inflammatory makers during exacerbation [143, 144].

Secondary bacterial infection, particularly *Haemophilus influenzae* [143, 144], is detected in 60 % of subjects with COPD after rhinovirus infection compared with 10 % in healthy smokers and nonsmokers. Sputum neutrophil elastase was significantly increased and SLPI and elafin levels significantly reduced after rhinovirus infection exclusively in subjects with COPD with secondary bacterial infections with SLPI and elafin levels correlating inversely with bacterial load [143, 144].

The role of the lung microbiome in the immunopathology of COPD remains unknown although the bacterial load of the bronchial mucosa of patients with stable COPD is related to the intensity of the airway inflammation and to disease progression [133]. Chronic bacterial colonisation could contribute to the increased susceptibility of COPD patients to viral infection, for example, by increasing ICAM-1 expression on the surface of the bronchial epithelial cells [145].

The development of such human experimental model in which causation is clearly defined and in which detailed clinical, immunological and inflammatory studies on the mechanisms of COPD exacerbations can be carried out, will offer an invaluable tool to increase our understanding of the specific COPD immunopathology during exacerbations induced by rhinovirus.

### Limitations of studies in COPD immunopathology

Small airway specimens are most frequently obtained from subjects undergoing lung resection for peripheral carcinomas or from subjects with pulmonary emphysema undergoing lung volume reduction surgery (LVRS). The selection criteria create important biases in the analysis of inflammation of the airways and parenchyma. Of utmost importance is the need to compare the results obtained from COPD patients with age-matched control subjects.

The lung tissue specimens from lung resection for peripheral carcinomas came mainly from patients with nonsmall cell lung cancer (NSCLC). Inflammation in the neoplastic lungs of patients with NSCLC in variable in the different studies, but usually there is a predominance of Th1 cells independent of concomitant COPD severity [146] and of Tregs [147]. Furthermore, cancer cells may also suppress dendritic cell maturation [148]. To minimise these concerns, it is important to obtain the non cancerous lung tissue as far as possible from the primary lung lesion.

Studies on the pathology of COPD bronchi have contributed significantly to our current knowledge, but an important limitation, particularly those in patients with severe disease, is a potential effect of steroid treatment. Although not generally a problem with peripheral airway samples obtained by lung resection for peripheral carcinomas as these patients are not usually treated with ICS, this is an issue with severe and very severe COPD samples obtained by LVRS as these patients have almost invariably been treated with high-dose ICS and/or other drugs and are often infected by bacteria. In a similar manner, smoking status and use may affect the histopathological features observed. Ideally, it is important to have biochemical proof of smoking status.

The comparison of results over time has been complicated by the fact that the definition of COPD in both National and International guidelines has changed many times. When examining clinical lung function data presented in some of the older studies (for example, see Table 1 in [149]), some patients with COPD or control smokers with normal lung function should be reclassified to a different clinical phenotype.

Finally, there are some methodological issues that need to be controlled in that most studies use histochemical and immunohistochemical techniques which require unmasking of the epitope in paraffin sections and not all antibodies work well under these conditions and detection of some markers are suboptimal using high-throughput analysis. Gene expression studies performed using microarrays or next-generation sequencing provides enormous amounts of information but lack precision as to cellular origin. However, recent bioinformatic approaches have enabled cell subsets to be much better defined using gene signatures.

## Conclusions

The immunopathology of COPD is based on the innate and adaptive inflammatory immune responses to the chronic inhalation of cigarette smoking. In the last quarter of the century, the analysis of specimens obtained from the lower airways of COPD patients compared with those from a control group of age-matched smokers with normal lung function has provided novel insights on the potential pathogenetic role of the different cells of the innate and acquired immune responses and their pro/anti-inflammatory mediators and intracellular signalling pathways, contributing to a better knowledge of the immunopathology of COPD both during its stable phase and during its exacerbations. The COPD immunopathology is largely driven by a complex cross-talk between macrophages and dendritic cells and lymphocytes which trigger both cell-mediated and antibody-mediated chronic inflammation and remodelling of the lower airways with the clinical consequences of irreversible airflow limitation and respiratory symptoms. This process is likely triggered by many components of the tobacco smoke especially oxidative/nitrosative/carbonyl stress. Greater understanding of the pathophysiology of COPD will provide an informed basis for rational drug development.
